# Simple Method of Home Modification of Milk and Its Application in Infant Feeding

**Published:** 1904-09

**Authors:** Louis C. Rouglin

**Affiliations:** Atlanta, Ga.


### SIMPLE METHOD OF HOME MODIFICATION OF MILK AND ITS APPLICATION IN INFANT FEEDING.

BY LOUIS C. ROUGLIN, M.D.,
ATLANTA, GA.

To the physician of whom the care and charge of the growing child is required, the problem of feedings to fit forever recurs. A mother's milk may or may not meet the requirement of her own offspring. When the mother's milk fails, it usually means a poor milk and does not meet the requirement of the child. A wet nurse may or may not be a fit one. The normal child is not the same infant in its physiologic demands more than a few weeks at a time. A growing child is not the same child for more then two weeks at a time, it has its ever-changing needs and the feedings should be re-adjusted to its growth—in other words, the feedings should keep pace with the ever-changing physiological condition of the child they should fit.

In substitute feeding let us assume:

1. That cow's milk is the only variety of milk to be considered.
2. That the milk must be clean and fresh.
3. That it must be modified according to a definite formula.
4. That the modification must vary according to the needs of the growing and ever-changing infant.

Modification of milk then consists in the transformation of the proportions of cow's milk to the proportion of woman's milk and in transforming a slightly acid milk to one slightly alkaline in reaction, in preserving it from bacterial and toxic contamination, in holding it ready in divided quantities for the periodical requirements of the infant—in short, making a substitute as nearly as possible as mother's milk and to administer same in proper quantities according to the demands of the child.

The home modification of milk on a percentage basis as introduced by Dr. Rotch has never come into general use owing to the fact that the method of obtaining such percentage modifications has

---

*Read before the Atlanta Medical Society Thursday August 4, 1904.

never been made so simple as to be readily understood by busy physicians. The methods by algebraic formulary, while good and accurate, involve study and practice, while the decimal and all methods involving the use of milk-sugar solutions are complicated and does not readily appeal to the general practitioner only a part of whose practice consists in feeding infants. As a result of such methods it has led to the inference of difficulty in obtaining exact percentages at home.

I believe such inference to be entirely erroneous and think it is desirable to bring clearly before the profession a simple mathematical method of determining such percentages at home without formula —The method I am about to describe is one advocated and taught by Dr. Rowland Freeman of New York, and I can from personal experience testify to its simplicity and efficiency—in order to do so, the physician should have a knowledge of the composition of mother's milk, cow's milk, and what is usually used in connection with same, whey and barley water; also about the percentages of fat in different kind of creams and what percentages it is necessary for a child as it grows and changes in its demands.

### METHODS OF PREPARATIONS.

Whey is obtained by heating cow's milk to a warm temperature and adding to each pint, one dram of Fairchild's Essence of Pepsin. This will divide the milk into junket and whey. The whey contains approximately:

*Table No. 1. Formula for Whey.*

| | |
|---|---|
| Proteids | 0.86 |
| Fat | .32 |
| Sugar | 4.79 |
| Salts | .65 |
| Water | 99.38 |

*Method for Preparing Barley-water.*

Take half an ounce of barley to a pint of water and boil for six hours—Robinson's barley requires only one hour boiling. This contains:

*Table No. 2. Formula for Barley Water.*

| | |
|---|---|
| Starch | 1.63 |
| Fat | .05 |
| Proteids | .09 |
| Inorganic salts | .03 |
| Water | 98.20 |

Now as to milk, the physician should know that average cow's milk contains approximately:

*Table No. 3. Formula for Cow's Milk.*

| | |
|---|---|
| Fat | 4% |
| Sugar | 4% |
| Proteids | 4% |

and that woman's milk contains,—

*Table No. 4. Formula for Woman's Milk.*

| | Low average. | High average. |
|---|---|---|
| Fat | 3% | 4% |
| Sugar | 6% | 7% |
| Proteids | 1% | 2% |

and that cream especially which forms on milk after it has stood in a refrigerator for twelve hours contains,—

*Table No. 5. Formula for 12-hour Cream.*

| | |
|---|---|
| Fat | 16% |
| Sugar | 4% |
| Proteids | 4% |

Having determined upon the chemic composition of the milk desired to use in any particular case, the number of feedings, and the amount to be given at each feeding, he must first note the relationship that exists between the fats and proteids—that is, whether the fat should be in equal amount to the proteid, or should it be in relation of two to one, three to one, or four to one, and arrange your formula so that one of these four relationships between fats and proteids may be present. This affords sufficient variety in formulas, and adds considerably in simplicity in obtaining the modification desired.

If he wishes to use the fat in equal proportions to the proteids, the relation in ordinary cow's milk is then suitable, when a 2% fat and proteid is desired a dilution of this with equal parts of water will give the desired proportion.

*Table No. 6. Method of Obtaining a 2 per cent. Cream and Proteids.*

| | Fat. | Sugar. | Milk. |
|---|---|---|---|
| 1 part cow's milk | 4 | 4 | 4 |
| 1 part water | 0 | 0 | 0 |
| | 2)4 | 4 | 4 |
| | 2 | 2 | 2 |

If a 1 per cent. fat and proteid is desired, dilution with three parts of water will give the desired formula.

*Table No. 7. Method of Obtaining a 1 per cent. Fat and Proteid.*

| | Fat. | Sugar. | Milk. |
|---|---|---|---|
| 1 part cow's milk | 4 | 4 | 4 |
| 3 parts water | 0 | 0 | 0 |
| | 0 | 0 | 0 |
| | 0 | 0 | 0 |
| | 4)4 | 4 | 4 |
| | 1 | 1 | 1 |

If on the other hand it is desirable to have a dilution containing four times as much fat as proteids, it is evident that a 16 per cent. cream properly diluted will furnish the required proportion.

*Table No. 8. Formula for 16 per cent. Cream.*

| Fat. | Sugar. | Proteid. |
|---|---|---|
| 16 | 4 | 4 |

If three times as much fat as proteids is required, we must have a 12 per cent. cream, which is easily obtained by taking two parts of 16 per cent. cream and one part milk.

*Table No. 9. Method of Obtaining a 12 per cent. Cream.*

| | Fat. | Sugar. | Proteid. |
|---|---|---|---|
| 2 parts 16 per cent. cream | 16 | 4 | 4 |
| | 16 | 4 | 4 |
| 1 part cow's milk | 4 | 4 | 4 |
| | 3)36 | 12 | 12 |
| | 12 | 4 | 4 |

If the fat is twice the amount of the proteid it is evident that we must have an 8 per cent. cream, which is obtained by mixing one part of 16 per cent. cream with two parts of milk.

*Table No. 10. Method of Obtaining an 8 per cent. Cream.*

| | Fat. | Sugar. | Proteid. |
|---|---|---|---|
| 1 part 16 per cent. cream | 16 | 4 | 4 |
| 2 parts milk | 4 | 4 | 4 |
| | 4 | 4 | 4 |
| | 3)24 | 12 | 12 |
| | 8 | 4 | 4 |

Thus it can be readily seen that by the different dilutions of gravity, cream and milk, most any percentage of cream desired may be obtained; for instance, should a 10 per cent. cream be desired, same can be obtained by mixing equal parts of gravity cream with milk.

*Table No. 11. Method of Obtaining a 10 per cent. Cream.*

| | Fat. | Sugar. | Proteid. |
|---|---|---|---|
| 1 part 16 per cent. cream .............. | 16 | 4 | 4 |
| 1 part milk ............................ | 4 | 4 | 4 |
| | 2)20 | 8 | 8 |
| | 10 | 4 | 4 |

And by diluting these with different parts of water a great variety of modifications may be obtained.

Now as to the requirements of the infant at its different periods of growth, the following table will be of service.

*Table No. 12. Modifications Required for Infants Feeding and How to Admimister Same.*

| Age. | Fat. | Sugar. | Proteids. | Amount at Each Feeding. | Interval of Feedings. | No. of Feed-ings 24 H'rs. | No. of Night Feed-ings. |
|---|---|---|---|---|---|---|---|
| 1/2 days. | 0- | 4- | 0 | 1- ounce. | 2 hours. | 10 | 1 |
| 3/7 days. | 2. | 6 | 0.6 | 1- " | 2 " | 10 | 1 |
| 1/4 weeks. | 2. | 6 | 1. | 1 " | 2 " | 10 | 1 |
| 1/2 months. | 3. | 6 | 1. | 2-3 " | 2½ " | 9 | 1 |
| 3/6 " | 4. | 7. | 2.5 | 4-6 " | 3 " | 6 | 0 |
| 6/8 " | 4 | 7 | 3 | 6-7 " | 3 " | 6 | 0 |
| 9/12 months | 4 | 4 | 4 | 8 " | 3 " | 5 | 0 |

Assuming the above to be the scheduled formula for a normal child, the same child when sick should have a feeding appropriate for a younger child. In other words, reduce the strength of food for all sick children as in cases of intestinal indigestion, or when a child passes curds; place the baby first on barley water, then on whey, and gradually add cream or milk to the whey.

Increase the strength of feedings just as fast as the child can take it. The digestion of a healthy, growing child should be guided by its passages. When the passages become pasty—homogeneous—all one color, smooth like butter, canary-colored, an increase in the strength of the feeding is warranted. The fats, sugar and proteids should be advanced together, usually by a fraction of a hundred and at frequent intervals. A good, healthy infant may, to its advantage, be seen every two weeks and the feedings readjusted to its growth; in other words, the feedings should fit.

Now, suppose we desire to feed a healthy infant, two weeks old. We require ten feedings a day of two ounces each—twenty ounces—and it should contain 2 per cent. fat, 6 per cent. sugar, and 1 per cent. proteid, it is evident that an 8 per cent. cream mixed with three parts of water will meet our requirements, and same is prepared as follows:

*Table No. 13. Method of Obtaining Ten Two-Ounce Bottles of Modified Milk Containing 2 per cent. Fat, 6 per cent. Sugar, and 1 per cent. Proteid.*

| | Fat. | Sugar. | Proteids. | | | |
|---|---|---|---|---|---|---|
| ¼ 8% cream | 8 | 4 | 4 | | = 5 ounces | 16% cream 1⅔ oz.; milk 3⅓ oz. |
| | 0 | 0 | 0 | | | |
| ¾ water | 0 | 0 | 0 | | =13 | |
| | 0 | 0 | 0 | lime | | |
| | 4)8 | 4 | 4 | water | 2 | |
| | 2 | 1 | 1 | 10% | 20 ounces | |
| Milk, sugar, add | | 5% | | 5% of | 20 – 1 ounce. | |
| | 2 | 6 | 1 | | | |

Thus we have the desired formula except in the case of sugar which is 1 per cent. instead of 6 per cent. It is evident that 5 per cent. of sugar must be added. The amount of sugar necessary will thus depend upon the amount of milk used. This can be most conveniently ordered from the druggist in powders, one of which suffices for a day's food.

The above formula to the mother will read as follows:

| | | |
|---|---|---|
| 12 hr or 16% cream | 1⅔ | ounce. |
| Milk | 3⅓ | " |
| Water | 13 | " |
| Lime-water | 2 | " |
| Milk-sugar | 1 | " |

Suppose we have a child of fourteen weeks and we desire to give it eight feedings a day, from three to four ounces at each feeding, and it should contain 3 per cent. fat, 6 per cent. sugar, and 1 per cent. proteids, it is evident that a 12 per cent. cream diluted with three parts of water will give us our desired formula.

*Table No. 14. Method of Obtaining Eight Bottles, Four Ounces Each of Modified Milk Containing 3 per cent. Fat, 6 per cent. Sugar, 1 per cent. Proteid.*

| | Fat. | Sugar. | Proteid. | | |
|---|---|---|---|---|---|
| ¼ 12% cream | 12 | 4 | 4 | | = 8 oz. { 16% cream 5⅓ oz. / milk 2⅔ oz. |
| ¾ water | 0 | 0 | 0 | | |
| | 0 | 0 | 0 | | 22.4 ounces |
| | 0 | 0 | 0 | lime | |
| | 4)12 | 4 | 4 | water | 1.6 " |
| | 3 | 1 | 1) | 5% | 32 " |
| Sugar of milk, add | | 5% | | 5% of | 32 - 1.6 ounce. |
| | 3 | 6 | 1 | | |

This to the mother will read:

| | |
|---|---|
| 12 hr or 16% cream | 5⅓ ounces. |
| Milk | 2⅔ " |
| Water | 22.4 " |
| Lime-water | 1.6 " |
| Milk-sugar | 1.6 " |

Now, if the child is seven months old, we desire to give it six feedings a day, each feeding to contain from four to six ounces and to contain 4 per cent. fat, 7 per cent. sugar, 2 per cent. proteid, again an 8 per cent. cream diluted with equal parts of water will meet our requirement.

*Table No. 15. Method of Obtaining Six Bottles, Six Ounces Each of Modified Milk Containing 4 per cent. Fat, 7 per cent. Sugar, 2 per cent. Proteid.*

| | Fat. | Sugar. | Milk. | | |
|---|---|---|---|---|---|
| 1 part 8% cream | 8 | 4 | 4 | = 18 ounces | { 16% cream 6 oz. / milk 12 oz. / Water 16.2 oz. |
| 1 part water | 0 | 0 | 0 | = 18 " | Lime-water 5% 1.8 oz. |
| | 2)8 | 4 | 4 | | |
| | 4 | 2 | 2 | | |
| Sugar of milk, add | | 5% | | 5% of 36 - 1.8 ounce. | |
| | 4 | 7 | 2 | | |

This formula to the mother will read:

| | | |
|---|---|---|
| 12 hr or 16 % cream | 6 | ounces. |
| Milk | 12 | " |
| Water | 16.2 | " |
| Lime-water | 1.8 | " |
| Milk-sugar | 1.8 | " |

The above three formulas are necessary to remember, and these modifications should be moved gradually and frequently from one to another, so that at the age of nine months the proportions should approximate those of whole cow's milk; in other words, the child is ready then to be weaned.

SUMMARY.

This method of obtaining percentages in modifying milk is simple, can be conveniently done at home, and is applied as follows:

1. Having decided the number of feedings for twenty-four hours, the amount of each feeding, and the formula of food required, determine the desired relation between the fats and proteids and obtain a cream or milk in which these constituents exist in that proportion. Dilute this with the required amount of water, and add the required amount of milk sugar for twenty-four hours. If lime-water is added, deduct the amount of lime-water from the amount of water used.

2. Begin all feedings of new-born and sick infants with a very low percentage and increase the strength of the feedings as fast as the child can take it. The passages of the child should act as the guide for its required feeding.

3. At the age of four to six months add a teaspoon of orange or beef juice to the food to prevent scurvy.

4. The feedings should be frequently revised—frequent small percentages of increase in the fats, sugars and proteids are to be advised. A baby is not the same for many weeks at a time. Its growth and physiologic demands require frequent change of food.

5. At nine months the child should be practically weaned. Keep the child on milk during the first year, and on almost all milk during the second year.

*809–811 English-American Building.*

